# Identifying and Visualizing Macromolecular Flexibility in Structural Biology

**DOI:** 10.3389/fmolb.2016.00047

**Published:** 2016-09-09

**Authors:** Martina Palamini, Anselmo Canciani, Federico Forneris

**Affiliations:** The Armenise-Harvard Laboratory of Structural Biology, Department of Biology and Biotechnology, University of PaviaPavia, Italy

**Keywords:** structural biology, molecular recognition, protein flexibility, nuclear magnetic resonance, Small-angle scattering, X-ray crystallography, ensembles, cryo-electron microscopy

## Abstract

Structural biology comprises a variety of tools to obtain atomic resolution data for the investigation of macromolecules. Conventional structural methodologies including crystallography, NMR and electron microscopy often do not provide sufficient details concerning flexibility and dynamics, even though these aspects are critical for the physiological functions of the systems under investigation. However, the increasing complexity of the molecules studied by structural biology (including large macromolecular assemblies, integral membrane proteins, intrinsically disordered systems, and folding intermediates) continuously demands in-depth analyses of the roles of flexibility and conformational specificity involved in interactions with ligands and inhibitors. The intrinsic difficulties in capturing often subtle but critical molecular motions in biological systems have restrained the investigation of flexible molecules into a small niche of structural biology. Introduction of massive technological developments over the recent years, which include time-resolved studies, solution X-ray scattering, and new detectors for cryo-electron microscopy, have pushed the limits of structural investigation of flexible systems far beyond traditional approaches of NMR analysis. By integrating these modern methods with powerful biophysical and computational approaches such as generation of ensembles of molecular models and selective particle picking in electron microscopy, more feasible investigations of dynamic systems are now possible. Using some prominent examples from recent literature, we review how current structural biology methods can contribute useful data to accurately visualize flexibility in macromolecular structures and understand its important roles in regulation of biological processes.

## Introduction

Nearly all known biological processes require precise and often highly regulated interactions among macromolecules to exert macroscopic events including signal transduction, metabolism, tissue homeostasis, immune responses, and development. To perform their functions, biomolecules can adopt a multitude of conformations, including highly dynamic states and excited transition intermediates essential for enzymatic catalysis, signaling regulation, and protein–protein interactions (Petsko and Ringe, 1984; Vucetic et al., 2003; Eisenmesser et al., 2005; Lindorff-Larsen et al., 2005; Levitt, 2009; Motlagh et al., 2014; Chakravarty et al., 2015). The extent of the motions enabling these functions ranges from conformational changes limited to few angstroms displacements of side-chain rotamers (Fraser et al., 2009), to larger motions involving flexible stretches of amino acids (Qin et al., 1998; Williams et al., 2014), to broad subunit rotations involving molecular rearrangements of several nanometers (Bennett and Huber, 1984; Korostelev and Noller, 2007; Forneris et al., 2010; Menting et al., 2013).

A deep understanding of conformational variability in macromolecules is a fundamental step forward in our knowledge of key biological processes. Flexible regions are critical elements for recognition of macromolecular interactions, and acquire even more fundamental roles when modifications altering the binding kinetics and/or affinity alter the overall biological significance of such interactions (Lim, 2002; Ekman et al., 2005; Levitt, 2009; Forneris et al., 2012). A valuable example is provided by the molecular recognition displayed in numerous epigenetic regulators of post-translationally modified histone tails, frequently resulting in opposite gene expression states depending on the readout of the specific histone tail reader or modifier involved (Bowman and Poirier, 2015; DesJarlais and Tummino, 2016; McGinty and Tan, 2016). Other paradigmatic examples include the conformational changes displayed by receptor tyrosine kinases during ligand-mediated activation of signaling cascades (Menting et al., 2013; Nikolov et al., 2013), or the flexibility between Fc and Fab regions in immunoglobulins, critical for antigen recognition (Tainer et al., 1984; Lilyestrom et al., 2012).

Given their elusive nature, dynamic processes are amongst the most difficult to characterize. Molecular flexibility often remains obscured in structural biology research, as demonstrated by our limited structural knowledge of events such as protein folding, allosteric mechanisms, as well as the difficulties in the characterization of intrinsically disordered proteins (Vucetic et al., 2003; Wright and Dyson, 2009; de Amorim et al., 2010; Motlagh et al., 2014; Kachala et al., 2015). Nevertheless, the importance of understanding the precise contributions of flexibility in macromolecular systems has long been recognized by the structural biology community (Petsko and Ringe, 1984; Rejto and Freer, 1996; Wilson and Brunger, 2000; Levitt, 2009; Tompa et al., 2014; Woldeyes et al., 2014).

Contemporary methods can provide very useful, but still limited, concepts regarding dynamically random systems such as intrinsically disordered proteins (Vucetic et al., 2003; Bernadó and Svergun, 2012; Kachala et al., 2015). On the other hand, the investigation of flexibility associated to conformational changes can highly benefit from the latest methodological advances in structural biology. For example, very recent studies using cryo-EM are now providing descriptions of molecular architectures and functions that were barely imaginable a few years ago (Kühlbrandt, 2014; Bai et al., 2015a; Callaway, 2015; Merk et al., 2015). Next to the advances in cryo-EM, characterizations of transiently interacting systems using crystallography and solution techniques also contribute crucial details on how conformational changes often enable unpredictable intermolecular contacts, generating specific binding platforms for ligand binding and/or catalysis (for recent examples, see Forneris et al., 2010; Rasmussen et al., 2011; Menting et al., 2013; Dong et al., 2016; Thach et al., 2016). Analysis of these results often highlights how our current understanding of biological mechanisms suffers the limitations of conventional “*single model”* structural characterizations, lacking fundamental regulation aspects frequently mediated by allostery or conformational dynamics.

The outcome of a successful structural biology study is a resolution-dependent three-dimensional representation of the molecular architecture of the system of interest, accurately reconstructed from the experimental data with the help of computational tools. In general, the investigation focuses on well-folded macromolecules, usually homogeneously purified in non-native conditions. The resulting characterization (and the related investigation of molecular flexibility) is necessarily influenced by the technique of choice. Depending on the approach, sample preparations include a variety of buffer solutions, crystals, vitreous ice, or heavy atom staining, which may severely impact on the nature of the intrinsic dynamics and interactions displayed by macromolecules. Furthermore, using techniques such as crystallography or cryo-EM, interpretation artifacts may arise from trapping the molecules inside three-dimensional crystal lattices or vitreous ice, respectively (Isenman et al., 2010; van den Elsen and Isenman, 2011). Sample preparation conditions for solution studies are usually more gentle, however techniques such as biological NMR require isotope labeling and high sample concentrations, which are anything but physiological and may be as prone to artifacts as crystallography or cryo-EM (Clore et al., 1994, 1995).

In many cases, structural models only implicitly include data about protein dynamics and conformational heterogeneity. Such information is often inferred by the absence of interpretable electron density from X-ray diffraction and electron microscopy data, by a limited number of distance/orientational restraints in nuclear magnetic resonance (NMR), or by lack of detailed features in small-angle X-ray scattering (SAXS) curves, usually indicating multiple co-existing conformations or oligomeric states in solution (Pelikan et al., 2009; Bernadó, 2010; Fenwick et al., 2014; Lang et al., 2014; Rawson et al., 2016). Despite providing clear indications for the presence of molecular flexibility, these implicit information do not enable visualization and understanding of the physiological roles of dynamics in the biological system of choice, or their possible contributions to molecular recognition (Burnley et al., 2012; Lang et al., 2014; Woldeyes et al., 2014). Furthermore, even when detailed time-resolved studies are achievable (Schmidt et al., 2004; Doerr, 2016), understanding the physiological time correlation between the various recorded states remains a challenge (Schmidt et al., 2004; Woldeyes et al., 2014; Correy et al., 2016). For example, mapping the allosteric continuum of functional conformations involved in ligand binding and downstream signaling in highly dynamic G protein-coupled receptors is still experimentally unreachable (Westfield et al., 2011). It's like watching isolated frames of a movie without knowing exactly how to connect the various scenes.

Here, we review the most recent developments in experimental investigation of dynamics and flexibility using structural biology, focusing on examples related to molecular recognition. Given the very large number of outstanding three-dimensional structures published every week, we do not aim to provide a comprehensive overview of the literature. Instead, we try to shed light on a few recent cases that, in our opinion, effectively illustrate the usage of conventional and modern structural biology techniques to visualize molecular flexibility and understand its biological functions. By also increasing the appetite toward incoming near-future developments of structural biology investigation, we hope that our work will inspire more researchers to consider this relatively poorly explored field.

## Crystal structures offer more than isolated static conformations

### Crystal structures and flexibility: is *B* factor analysis sufficient?

Over the last half-century, X-ray crystallography has been the most used and useful methodology to elucidate three-dimensional structures of biological macromolecules. The investigation of protein dynamics using X-ray diffraction is not novel (Petsko and Ringe, 1984); however, for studies involving molecular flexibility, crystallography is likely one of the least considered approaches to tackle such challenges. Most entries in the Protein Data Bank (PDB) (Berman et al., 2003) derived from X-ray diffraction data are presented as static, conformationally averaged structural models regularly trapped in a three-dimensional lattice. However, even in crystal structures proteins are all but rigid, and constantly sample conformational substates that may be highly relevant for their biological functions (Frauenfelder et al., 1991; Fenwick et al., 2014; Xue and Skrynnikov, 2014; Ma et al., 2015). This is confirmed by exploring dynamics in X-ray diffraction datasets collected at different temperatures (i.e., from crystals frozen in liquid nitrogen and at room temperature (RT)). RT crystallography experiments, although much more sensitive to radiation damage, can indeed provide extensive information about molecular motions in a nearer physiological environment than at liquid nitrogen temperatures (Fenwick et al., 2014; Woldeyes et al., 2014).

Information about molecular motions is incorporated inside X-ray crystal structures through *B* factors, which represent temperature-dependent vibrations from average atomic positions (García et al., 1997). Depending on the resolution, *B* factors may parameterize thermal motions associated to individual atoms, isotropically or anisotropically. *B* factors essentially quantitate the uncertainty of atomic positions, and include convoluted information about molecular flexibility, crystalline disorder, discrepancies between model and data, as well as the quality of structural refinement. However, dissecting the individual contributions of these elements to *B* factors is not possible (Vitkup et al., 2002; Kuzmanic et al., 2014). Pure *B* factor analysis may thus lead to inaccurate interpretation of molecular flexibility, particularly when the end users are non-crystallographers (Wlodawer et al., 2008). A translation, libration and screw (*TLS*) model can additionally account for anisotropic deviations for groups of atoms identified based on their involvement in molecular motions. Each atom of the group is approximated as part of an ideal rigid body that is displaced normally about a mean position (Winn et al., 2001; Urzhumtsev et al., 2015). Analysis of anisotropy of the various *TLS* groups in a PDB file can provide an additional layer of information about molecular flexibility, complementing the atomic *B* factors. *TLS* analysis often highlights domain motions in large systems (Mouilleron and Golinelli-Pimpaneau, 2007), or local rearrangements of highly flexible motifs inside enzyme catalytic sites (Tanner et al., 1993), or highly flexible solvent-exposed regions of macromolecules (Van Benschoten et al., 2015).

Regions with weak or non-interpretable experimental electron density due to high flexibility are usually modeled with a single conformer with elevated *B* factors, or not modeled at all (Schneidman-Duhovny et al., 2014; van den Bedem and Fraser, 2015). Besides the complexity associated to the significance of *B* factors and *TLS* components in measuring flexibility in crystal structures, it is now broadly accepted that *B* factors *per se* overall underestimate molecular motions (Vitkup et al., 2002; Fenwick et al., 2014; Kuzmanic et al., 2014; Woldeyes et al., 2014). Such underestimation becomes particularly critical in highly dynamic regions (Janowski et al., 2013; Kuzmanic et al., 2014). Recently, it has been suggested that *TLS* models used during structural refinement may have the potential to highlight correlated motions in crystal structures (Urzhumtsev et al., 2013, 2015). However, as during refinement *TLS* groups do not correlate with each other, there may be several different combinations of *TLS* groups equally well fitting the electron density. For this reason, analysis of *TLS* groups used in structural refinement to detect correlated molecular motions is far from immediate and reliable (Urzhumtsev et al., 2015; Van Benschoten et al., 2015). Accurate determination of experimental diffuse X-ray scattering from macromolecular crystals may facilitate motion analysis using *TLS*, because different *TLS* models yield markedly different computationally predicted diffuse patterns (Pérez et al., 1996; Héry et al., 1998). Thus, accurate comparison of computed and experimental diffuse scattering patterns could allow discriminating between correlated and non-correlated variations in the electron density distributions, enabling identification of a *TLS* configuration representative of true molecular motions. The first tools to perform these computational analyses are nowadays available (Van Benschoten et al., 2015).

Numerous studies have emphasized the signatures of dynamics in crystallographic data, suggesting that the molecular motion details can be extrapolated from weak experimental electron densities much further than using simple thermal motion analysis (Lang et al., 2014; Woldeyes et al., 2014; Van Benschoten et al., 2015). Indeed, the presence of extensive disorder resulting from conformational heterogeneity and crystal-lattice distortions is frequently detectable (Kruschel and Zagrovic, 2009; Burnley et al., 2012; Ma et al., 2015). The weaker electron density regions include noise from experimental and model errors, but also convoluted details compatible with populations of alternative polypeptide and side-chain rotamers, and low-occupancy ligands. These multiple conformations are averaged across unit cells in space, and also within unit cells in time during the X-ray diffraction experiment (Levin et al., 2007; Terwilliger et al., 2007; Lang et al., 2014; Woldeyes et al., 2014; Van Benschoten et al., 2015). Separating the information about molecular flexibility in electron density maps from the noise due to experimental error and crystal lattice distortions holds massive potential, as it will facilitate enzyme inhibitor development and drug discovery, connect macromolecular motions to biological functions, and provide a visual support to molecular flexibility (Burnley et al., 2012; Lang et al., 2014).

### Dissecting molecular flexibility in crystal structures using ensemble refinement

**Figure 1:**
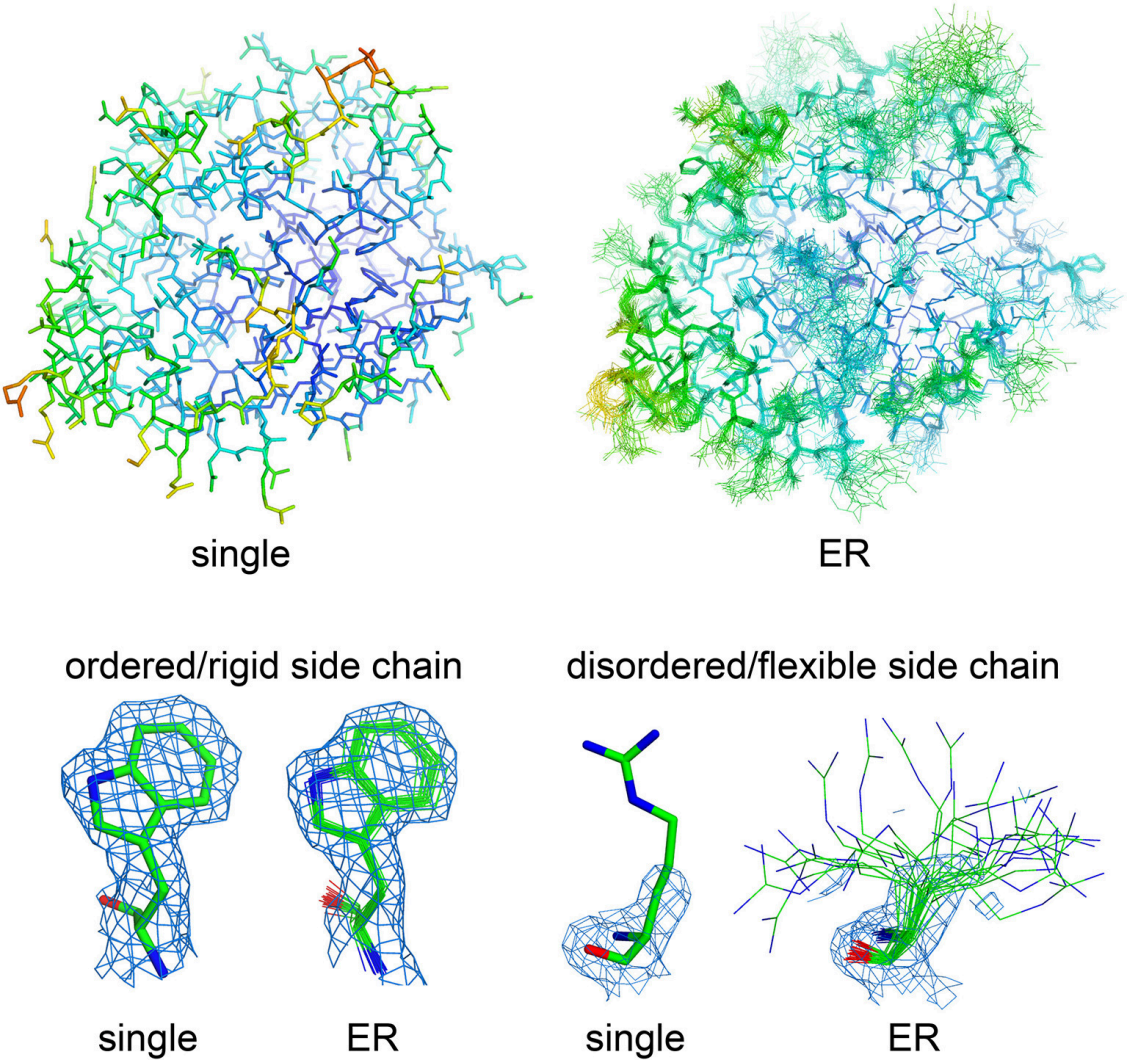
**Visualizing molecular flexibility using structural ensembles**. Ensemble refinement of macromolecular crystal structures: from a single, *B* factor-weighted static model to a superimposed “*bouquet*” of structural conformations, providing deeper understanding of local flexibility even inside the crystal lattice. The structural models (represented as sticks) and electron density maps (blue mesh, 2F_o_–F_c_ maps contoured at 1.2 σ) for single- and ensemble-refined data were from PDB files 4CBN and 4CBO, respectively (Forneris et al., 2014). The structures are colored based on their isotropic atomic *B* factors, using the same scale from 10 (blue) to 100 (red) Å^2^. Figure prepared using *PyMol* (Schrödinger, LLC, 2010).

How can we accurately extrapolate the true structural diversity of biomolecules from X-ray diffraction data, without the risk of misleading interpretations? Multiple strategies have been developed over the last 20 years, but due to technical complexity, limitations in applicability, and initial methodological failures, they never spread broadly throughout the structural biology community. The common theme of these methods is that distributions of molecular conformations (similar to NMR ensembles) may provide more accurate and complete representations of a protein's native state also in crystal structures (Best et al., 2006; Levin et al., 2007; Terwilliger et al., 2007; van den Bedem et al., 2009; Tyka et al., 2011; Burnley et al., 2012; Woldeyes et al., 2014; Xue and Skrynnikov, 2014; Clark et al., 2015). Two main strategies allow generation of molecular ensembles from X-ray datasets, time-averaged (Burnley et al., 2012) and multiconformer (van den Bedem et al., 2009) ensemble refinement (ER). Both methods fit the experimental electron density better than a single structural model, without overfitting the data as occurred with original developments of time-averaged ER (for a recent review on ER methods, please see Woldeyes et al., 2014). In time-averaged ER, generation of multiple conformers is assisted through X-ray data-restrained molecular dynamics (MD) simulations, which generate optimal superpositions of a subset of structural models that fit the electron density. The procedure automatically restricts the final number of conformations in the ensemble models by running short MD simulations (0.25–2 ps), preventing data overfitting. Critical parameters for ensemble refinement are the relaxation time of the simulation (which depends on data resolution) and the percentage of atoms used for *TLS* grouping (Burnley et al., 2012). Usually, these two parameters are determined empirically through parallel ER runs, by selecting the combination which yields the best refinement statistics (based on *R*_*work*_/*R*_*free*_ values; Burnley et al., 2012; Burnley and Gros, 2013). In multiconformer ER, the selection of the optimal number of conformations for each segment of the molecule is based on how well each segment fits the experimental density (van den Bedem et al., 2009). Therefore, time-averaged ER structures include multiple models with the same number of states throughout the entire macromolecular sequence, whereas multiconformer ER models display a variable number of states within specific regions of the crystal structure, depending on the quality of the experimental electron density (Woldeyes et al., 2014). The result of ER is, therefore, a set of superimposed molecular models, more similar to the final output of an NMR structural investigation than a crystal structure, with increasing deviations from the average conformation for the highly flexible regions and nearly perfectly superimposed conformations in the more rigid portions of macromolecules (Figure 1). Flexible elements are shown with a “*bouquet*” of conformations, each one representing just a fraction of the total population that fits the poorly defined electron density of the highly dynamic region. However, when considered altogether, structural ensembles capture the multiple conformations displayed by various regions of the crystallized macromolecules, poorly represented and hard to understand by *B* factor analysis (Lang et al., 2014; Woldeyes et al., 2014). Even though these methods are not recent, their diffusion has so far been very limited, mostly due to the heavy computational resources that are needed to generate reliable ensembles (Burnley and Gros, 2013; van den Bedem and Fraser, 2015). However, there is strong interest about their potential as standalone methods for the investigation of structural dynamics, as demonstrated by the increasing number of publications making use of these strategies for the analysis of conformational flexibility (Fenwick et al., 2011; Forneris et al., 2014; Bianchetti et al., 2015; Weerth et al., 2015; Cao et al., 2016; Langan et al., 2016).

Examples of successful application of ER include the accurate analysis of flexibility in proline isomerase in diffraction datasets collected at different temperatures (Fraser et al., 2009; Burnley et al., 2012), ubiquitin (Fenwick et al., 2011; Burnley et al., 2012), dihydrofolate reductase (Fenwick et al., 2014) and thrombin allostery (Forneris et al., 2014), revealing functional features consistent with experimental biophysical characterizations in solution (Eisenmesser et al., 2005; Huntington, 2008; de Amorim et al., 2010; Lechtenberg et al., 2010; Fenwick et al., 2014). Using time-averaged ER on high resolution data collected from protease complement factor D (FD) crystals, it was possible to highlight dramatic conformational dynamics in regions where the electron density was poorly defined after conventional refinement. In this case, the ER analysis revealed an unprecedented aspect of FD biology, showing that this protease undergoes a highly flexible intermediate state during recognition and interaction with its macromolecular substrate. Such dynamics, purely observed inside a crystal structure (with fluctuations reaching 5–6 Å from average atomic positions in the most flexible areas), is reminiscent of thrombin allostery associated to ligand binding. However, in FD this flexible state is constrained between a remarkably rigid inactive state and a likewise rigid substrate-bound conformation, as observed in free and substrate-bound crystal structures (Narayana et al., 1994; Forneris et al., 2010, 2012, 2014).

It should be noted that experiment temperature, crystal packing contacts, and distortions in the crystal lattice will have a strong influence on the ER models and may affect the overall interpretation of structural dynamics. Therefore, ER users should keep in mind that, although very powerful, even in a perfect crystalline sample ER models will always capture the conformational dynamics of molecules constrained inside a crystalline state, which may differ strongly from what happens in solution (Fenwick et al., 2014; Woldeyes et al., 2014). Furthermore, ER methods only provide a better sampling and clearer visualization of what the experimental electron density is already showing. Very likely, non-interpretable highly disordered regions of the electron density will remain non-interpretable, and ensemble models will simply facilitate the visualization of such disorder and high flexibility. Analogous considerations should be made for low-resolution data (below 3 Å), where structural ensembles are unlikely to provide useful information (van den Bedem et al., 2009; Burnley et al., 2012; Burnley and Gros, 2013; Woldeyes et al., 2014).

### Adding a fourth dimension: time-resolved and kinetic crystallography

Time-resolved crystallography experiments using synchrotron radiation constitute an interesting although rather minor branch of structural biology focusing on structural dynamics (Bourgeois and Royant, 2005; Graber et al., 2011). Depending on the implemented methodology, methods such as pump-probe Laue diffraction and freeze-capture kinetic crystallography allow obtaining time resolutions from seconds to hundreds of picoseconds (Lindenberg et al., 2000; Schotte et al., 2004, 2012). These methods proved highly successful in exploring enzyme mechanisms and variations in reactive centers (Bourgeois and Royant, 2005; Kim et al., 2012). Pump-probe Laue crystallography is the traditional approach to time-resolved investigation. This technique combines collection of diffraction patterns from multi-wavelength X-ray pulses after triggering reactions within crystals, typically using a laser pump-pulsed source of X-ray, visible or infrared radiation (Spence et al., 2012). By performing experiments at different temperatures, specific induction of radiation damage or its control through freeze-trapping allow structural determination of intermediate enzymatic states, and understanding of conformational dynamics associated to the triggering event (Bourgeois and Royant, 2005).

These experiments are limited to dynamic biological systems where a specific triggering signal from the laser pump can be used to perform the pump-probe measurements. Furthermore, the time resolution offered by X-ray pulses at synchrotron sources represents another significant limitation, as it does not allow to capture conformational changes below picoseconds. However, recent developments suggest a broader range of applications of these methods even using conventional synchrotron sources, offering opportunities for time-resolved crystallography to a larger scientific community (Yorke et al., 2014).

For studies on highly flexible systems, room temperature crystallography is experiencing a new spring. The introduction of high-throughput data collection pipelines for frozen crystals at synchrotrons somehow lowered the interest toward this approach over the years. However, recent examples of synchrotron diffraction data collected at room temperature with careful control of radiation-induced damage illustrate the usefulness of this method in exploring molecular flexibility (Stellato et al., 2014; Levantino et al., 2015), even using very sensitive samples such as lipidic-cubic phase-grown crystals of integral membrane proteins (Nogly et al., 2015).

### Eliminating radiation damage effects using free electron lasers

A leap forward in understanding molecular flexibility and conformational variation in crystal structures is provided by serial femtosecond crystallography (Chapman et al., 2011). Using next generation X-ray free electron laser (XFEL) light sources, ultrashort, high intensity pulses can be used in “*diffraction before destruction*” experiments (Chapman et al., 2014), to collect high resolution single diffraction images from nanocrystals passing into the XFEL beam through a microspray system. The rapidity of the X-ray pulse immediately preceding crystal disintegration allows obtaining (after structure determination) three-dimensional snapshots of the crystallized molecule. Such setup is compatible with data collection of frozen crystals, as well as room temperature measurements. For a recent detailed review on the methodology, see (Martin-Garcia et al., 2016). With this approach, complete datasets can be obtained by exposing hundreds of thousands of randomly-oriented nanocrystals, collecting one single diffraction image before the high beam intensity disintegrates the crystal, and selectively “blending” the suitable diffraction images into a unique X-ray dataset for analysis.

Due to the femtosecond time scale of the X-ray pulses (shorter than the time required for radiation damage to occur) XFEL data are free of radiation damage (Chapman et al., 2011). Such rapid pulse is far beyond the achievable time resolutions used in conventional time-resolved studies at synchrotron sources (Cammarata et al., 2008; Levantino et al., 2015). Taken together, these features demonstrate how XFEL data collection can enable achieving radiation-damage free time resolutions that push the conventional limits of time-resolved data collection into the femtosecond time scale, enabling capture of ultrafast protein conformational changes which may remain completely elusive using more conventional sources (Liu et al., 2013; Keedy et al., 2015; Levantino et al., 2015; Doerr, 2016). As such, usage of XFEL offers the opportunity to study ultrafast conformational changes in the sub-picosecond time range, as already demonstrated by recent time-resolved studies of enzymatic mechanisms (Tenboer et al., 2014; Fukuda et al., 2016; Pande et al., 2016).

Considering the potentials of ensemble refinement and crystallography using XFELs, merging the two approaches for better understanding *in crystallo* dynamics appears as a very promising strategy. Such an idea has been exploited recently (Keedy et al., 2015), providing the first example of a conformational ensemble from XFEL data and suggesting exciting developments for the detection of concerted conformational changes upon ultrafast temperature changes, offering an opportunity to study correlated motions inside macromolecular crystals using ensembles.

## Analyzing conformational flexibility in solution

### Strengths and limitations of NMR analysis

Structural studies using NMR play a major role in understanding flexible systems and unstructured macromolecules (Wright and Dyson, 2009; Ravera et al., 2014; Dunker and Oldfield, 2015). X-ray crystal structures naturally complement such approach, by providing high-resolution information about conformationally stable fragments (Lindorff-Larsen et al., 2005; Huntington, 2008; Lechtenberg et al., 2010; Fenwick et al., 2014). Such combined analysis provides information about the time-scale of atomic motions, allowing better descriptions of the alternate conformational substates sampled through changes in picosecond-nanosecond time scales (Baber et al., 2001). However, the difficulties associated to NMR assignment of large macromolecular systems generally constitute a significant methodological limitation (Clore et al., 1994, 1995; Fenwick et al., 2014; Schwander et al., 2014; Clark et al., 2015). Approaches to overcome these limitations include the development of elegant strategies of selective side-chain isotope labeling (Otten et al., 2010) and development of long-distance NMR probes (Kato and Yamaguchi, 2015). These systems have provided valuable insights in flexibility of large systems, including the recent investigations on the extended motions associated to HSP90 chaperone function (Karagöz et al., 2011) and various molecular recognition events in the RNA polymerase complex (Drogemuller et al., 2015).

Next to NMR-specific developments, integrative approaches using advanced biophysics often allow bypassing the need for complex or poorly feasible labeling and assignment of NMR. These methods expand the research ground for scientists challenging flexibility in solution. Methods such as single molecule fluorescence energy transfer (Delaforge et al., 2015; Nagy et al., 2015), native and hydrogen-deuterium exchange coupled to mass spectrometry (Chen et al., 2010; Rostislavleva et al., 2015) often compensate the lack of interpretable information about molecular flexibility from direct NMR investigation. The increasing feasibility of computational simulations for large macromolecules is also significantly changing the field, offering larger room to integrative approaches merging structural predictions and advanced biophysical strategies next to more conventional structural techniques to explore molecular flexibility (Fenwick et al., 2014; Schröder, 2015; van den Bedem and Fraser, 2015).

### Solution scattering: valuable information at low resolution

With great improvements over the last years (Hura et al., 2009; Rambo and Tainer, 2010; Classen et al., 2013; Pernot et al., 2013; Dyer et al., 2014; Kachala et al., 2015; Kikhney and Svergun, 2015; Round et al., 2015; Tria et al., 2015), small-angle X-ray scattering (SAXS) and small-angle neutron scattering (SANS) (simply indicated as solution scattering techniques or SAS from now on) have turned from rather complex biophysical methods into high-throughput structural characterization techniques for complex macromolecular samples in their native state. Even though these methods provide very low resolution information compared to X-ray crystallography or modern cryo-EM, the structural details that can be reliably extracted from SAS experiments are very powerful for the analysis of conformation, shape, and dimensions of biopolymers ranging in size from short polypeptides to large viruses (Jacques and Trewhella, 2010; Dyer et al., 2014).

The main advantage in using SAXS for the analysis of macromolecules relates to the robustness and very rapid readout of various critical features of the sample, including homogeneity, size, low-resolution shape, molecular weight, stoichiometry and flexibility, even in absence of other structural information (for comprehensive reviews about the theoretical and practical aspects of these methodologies, please see Mitsui et al., 2007; Mertens and Svergun, 2010; Petoukhov and Svergun, 2013; Kikhney and Svergun, 2015). The low signal-to-noise readout of SAXS requires accurate sample preparation and very careful subtraction of the scattering contributions from buffering components, as well as excellent monodispersity (Mertens and Svergun, 2010; Kachala et al., 2015; Kikhney and Svergun, 2015). A prominent advance in facilitating sample preparation is the introduction of online size-exclusion chromatography systems immediately preceding the sample capillary at SAXS synchrotron beamlines (Pernot et al., 2013; Wright et al., 2013). This conceptually simple modification avoids most buffer subtraction issues and allows real-time selective separation of possible interfering components (oligomers, heterogeneous species) through the gel filtration matrix, increasing the chances of monodispersity and therefore more accurate measurements, directly at the beamline. Recent examples include analysis flexible therapeutic antibodies in various buffer environments (Tian et al., 2014) and the low resolution structural studies on large, heterogeneous proteoglycans (Watanabe and Inoko, 2013).

### Addressing flexibility using SAXS

Depending on sample quality and overall data resolution, the final outcome of SAS can be limited to the overall extent of macromolecules in solution (measured by the radius of gyration) or a finer description of macromolecular shape through *ab-initio* modeling and/or rigid body fitting (Jacques and Trewhella, 2010; Mertens and Svergun, 2010; Petoukhov and Svergun, 2013). The added value of SAS analysis is that these methods also directly provide useful data on flexible regions, expanding the investigation range of other high-resolution structural approaches (Classen et al., 2013; Kikhney and Svergun, 2015), also providing an effective complement to NMR studies of intrinsically disordered systems (Bernadó and Svergun, 2012; Dunker and Oldfield, 2015; Kachala et al., 2015). Flexibility has a dramatic effect on SAS data: it causes a general “*blurring*” and loss of detailed features in scattering curves (Bernadó and Svergun, 2012). Direct visualization of flexible features in SAXS samples can therefore be enhanced through accurate analysis of pair distance distribution function and Kratky plots (Bernadó, 2010; Rambo and Tainer, 2011). In particular, dimensionless Kratky analysis of SAXS data, typically used for investigating protein folding, provides a rapid yes/no result about the presence of conformational flexibility, allowing direct comparisons also among molecules differing in mass and conformational states (Rambo and Tainer, 2011; Bernadó and Svergun, 2012). More sophisticated methods, relying on Porod-Debye analysis of scattering decay, can further increase the reliability of the investigation and carefully distinguish intrinsic flexibility from conformational changes. These methods are more complex to apply, but when such analysis is possible, they efficiently allow discriminating between disorder associated to fully- or partially-unfolded states from flexible linkers connecting folded structural elements (Rambo and Tainer, 2011).

In flexible systems, the SAXS scattering profiles represent a weighted average over all the accessible conformations. *Ab-initio* models generated from these data may wrongly be fitted by single rigid-body structures, leading to data misinterpretation (Bernadó, 2010). Modern modeling techniques to circumvent such problems include, analogous to NMR and crystallography, generation of ensembles of conformers that fit the polydisperse sample (Bernadó, 2010; Bernadó and Svergun, 2012; Tria et al., 2015). Such a procedure is all but intuitive, because direct deconvolution of the contributions of each conformation to the overall SAXS curve is not possible (Kikhney and Svergun, 2015). Given the magnitude of molecular motions and multiple conformations that could be sampled in solution, it is critical to avoid overfitting of the data with too many states not representing the real conformational space (Pelikan et al., 2009; Kikhney and Svergun, 2015; Tria et al., 2015). At present, validation strategies to circumvent overfitting are still limited and rely on visual or computational inspection of the ensemble models, exclusion of inappropriate conformers and reduction of the overall pool of models into a feasible, but necessarily oversimplified, molecular ensemble (Bernadó et al., 2007; Pelikan et al., 2009; Hammel, 2012).

Nevertheless, ensembles originating from SAXS are excellent for the identification of interdomain motions in large multi-domain systems. In particular when high-resolution data from isolated domains is available, the knowledge gained from ensemble analysis can be crucial for the understanding of the biological function of the studied system as a whole, and the low-resolution information may provide guidance for important new investigations (Bernadó, 2010; Hammel, 2012; Tria et al., 2015). A remarkable example is offered by the analysis of how the small ubiquitin molecule binds to the PCNA interface in multiple states in solution. The SAXS analysis expanded the outcome of previous crystallographic studies, which showed only a single ubiquitin binding mode; such isolated conformation was likely selected by the crystal packing contacts. Solution studies revealed a whole range of motions possible for ubiquitin linked to PCNA that may have prominent roles in regulating ubiquitin-mediated DNA damage response (Tsutakawa et al., 2011). Similar *in crystallo* conformational selection was nicely shown in a recent publication about conformational variability of importin beta, illustrating how the environment surrounding the macromolecule may have a dramatic influence on quaternary structure and molecular flexibility (Tauchert et al., 2016). Another relevant case includes evidence for marked flexibility in the linker region of cytosine DNA methyltransferases that clarified previous controversies about their functional oligomeric states (Konarev et al., 2014).

### The added value of SANS

SANS can provide very useful, different, yet highly complementary information to SAXS analysis. While the principles of data analysis and interpretation are similar, SANS offers some advantage when combined with sample isotope labeling. Differences in neutron scattering intensity between hydrogen and deuterium can be used in contrast variation measurements (Gabel, 2012). This strategy proved useful to study complex systems, as shown for example in the SANS characterization of the intrinsic flexibility in apolipoprotein B-100 structure. SANS contrast variation allowed understanding the molecular features of a lipid-free apo B-100, allowing low resolution structural determination of a highly hydrophobic and flexible molecule, almost impossible to obtain using other methods (Johs et al., 2006).

Sample requirements and instrumental setups for SANS are however usually more technically challenging than SAXS, limiting its usage to combined SAXS-SANS experiments to maximize the information obtained from a sample in solution. Interesting examples of combined SAXS-SANS approaches include the determination of the architecture of neurexin-neuroligin interactions, important for synapse formation. In particular, using SANS contrast variation, it was possible to understand how two monomers of neurexin β are able to bind on opposite sides of the long axis of the neuroligin dimer in a defined orientation, a result which was validated by SAXS analysis and could not be obtained by X-ray crystallography due to the high flexibility of the macromolecular complex (Comoletti et al., 2007). Another example of synergy between SAXS and SANS measurements is the structural determination of drug-loaded liposomes. SANS allowed obtaining good contrast for the liposomal hydrophobic tails. Conversely, SAXS allowed studying the head groups. Depending on their hydrophobicity, different drugs interacted with the lipophilic tails or with the hydrophilic heads of the liposomes. The scattering profiles, measured using either SANS or SAXS, enabled accurate characterization of the interactions between the drugs and the particles (Di Cola et al., 2016).

### Using the power of XFEL to study dynamics in solution

Another fascinating recent development includes usage of solution wide-angle X-ray scattering (WAXS) at XFEL sources (Arnlund et al., 2014). In this method, the sample is flown through the intense pulsed XFEL source, providing conformational information at lower resolution than crystallography, but enriched of the ultrafast time resolution due to the femtosecond X-ray pulse. This methodology already showed promising results in probing ultrafast protein dynamics in light-sensitive protein centers (Cammarata et al., 2008; Takala et al., 2014; Levantino et al., 2015). Intriguingly, the combination of elements of WAXS analysis and time-resolved serial femtosecond crystallography seems to yield very promising results on studies of ultrafast conformational changes, further supporting the promise of obtaining time-correlated molecular movies from nanocrystals using XFEL sources (Aquila et al., 2012).

## The flexibility of mass spectrometry

Mass spectrometry (MS) is a very useful and versatile technique which allows not only to investigate the composition and overall mass of a macromolecular entity, but also to garner information on dynamics (Sharon and Robinson, 2007; Zhou and Robinson, 2014). While most MS approaches are inadequate for the mapping and visualization of protein flexibility, there are a few methodologies relying on soft ionization techniques (native mass spectrometry; Heck, 2008), which have allowed to do just that (Zhou and Robinson, 2014). The first of these involves coupling MS to a technique capable of highlighting regions of protein flexibility: hydrogen-deuterium exchange (HDX) (Wales and Engen, 2006). An example of synergistic analysis using HDX-MS and SAXS is provided by the investigation of large multidomain protein complement C3b and its conformational changes upon binding of bacterial ligands. These data showed how unprecedented flexibility and allosteric motions between folded states of C3b could be identified without high-resolution 3D structures of the complex available (Chen et al., 2010).

Coupling of chemical cross-linking to MS (XL-MS) offers a second possibility and provides information on spatial constraints between residues in a protein and/or subunits of a protein complex (Holding, 2015). This was employed, for example, to detail the inter-subunit interfaces and interactions of an F-type ATPase, evidencing a conformational shift dependent on the phosphorylation state of the protein complex. Such information was inferred by a change in spatial constraints investigated using a cross-linking agent. Integration of these data with pre-existing crystal structures, homology models, and crystal structures of homologous protein complexes, allowed describing this ATPase in a detail that might have been significantly hard to obtain with more conventional approaches (Schmidt et al., 2013).

A third approach using Ion Mobility (IM-MS) is particularly interesting as it can allow distinction between different conformers of proteins and protein complexes, as well as the generation of low resolution sphere models for previously unobserved structures (Ruotolo et al., 2008; Taverner et al., 2008). Structural models can then be further refined through computational methods such as MD, and/or by integration/comparison with homology models. While other low-resolution techniques like SAXS can provide similar data, the use of IM allows to isolate and interrogate individual conformers, a feature inaccessible to SAXS (Ruotolo et al., 2008). This experimental approach was used to investigate the conformational dynamics of a bacterial rotary V-type ATPase. IM-MS allowed interrogating the separate subdomains of this ATPase in different experimental conditions, thus highlighting structural shifts tied to the presence or absence of ATP. Moreover, it was possible to pinpoint the origin of the conformational heterogeneity to the flexibility displayed by the membrane subunit I of the ATPase V_0_ subdomain. Subsequent analysis of the IM-MS spectra of the other subdomains, integration with homology models and analysis of MD data through computational methods, allowed to evidence and model a continuum of conformations depicting the structural variations associated to its biological function (Zhou and Robinson, 2014; Zhou et al., 2014).

The potential of MS is elevated and the resolution of IM-MS is such that it can separate different protein conformers (Ruotolo et al., 2008; Zhou et al., 2014). However, most MS approaches allow only to infer data on structural flexibility and actual visualization is dependent on computational integration with pre-existing experimental data or homology models. Additionally, the best MS technique to provide *de novo* structural visualization (IM-MS) may suffer limitations due to instrument calibration and bias originating from reference models employed for the computational analysis (Ruotolo et al., 2008). Nonetheless these low resolution approaches often allow a more detailed investigation than many of their non-MS counterparts.

## Cryo-electron microscopy: unexpectedly quick advancements in structural biology

### The cryo-EM revolution

Nowadays, cryo-EM is a very powerful structural biology technique, as it combines the advantages of atomic resolution without the need for protein crystals, *de facto* overcoming the biggest bottleneck of protein crystallography and opening a whole new era of structural biology investigations (Kühlbrandt, 2014; Bai et al., 2015a; Subramaniam et al., 2016). The impact of this technique for understanding the molecular bases of biological processes, particularly in large macromolecular complexes, cannot be underestimated. Since 2013, the progress in cryo-EM has been so fast that it has been heralded as a *revolution* (Bai et al., 2015a; Callaway, 2015). Through major achievements in the methodology over the last years, including introduction of new direct electron detectors (DEDs) and improved image processing methodologies, it is now possible to obtain crystallography-comparable resolutions for macromolecules as small as 100–150 kDa using cryo-EM, even in absence of internal high-order symmetry as was indispensable until a few years ago (Allegretti et al., 2015; Bai et al., 2015b; Fernandez-Leiro et al., 2015). Furthermore, if the exciting promises offered by the new phase plate technologies are kept (Danev and Baumeister, 2016), cryo-EM will likely expand toward smaller molecular systems in the 50–100 kDa range and beyond (Merk et al., 2015; Subramaniam et al., 2016).

In cryo-EM the samples are rapidly flash-frozen in vitreous ice that preserves and stabilizes the aqueous environment of the sample in a native-like state. Such rapid process of vitrification also enables the trapping of transient states, often impossible to observe using X-ray crystallography (Kühlbrandt, 2014; Nogales, 2016; Thompson et al., 2016). During data acquisition, the electron beam may induce motions within the sample as well as radiation damage (Kühlbrandt, 2014). Whilst minimization of radiation damage is pivotal to successful structural determination using cryo-EM, the movement of the particle, also called beam-induced movement (BIM), may provide useful insights on the physiological dynamics of the molecules under characterization. Beam-induced movement affects the sample both spatially and temporally: distinct regions of the vitreous ice could show differential amounts of motion, which may also include large movements associated to flexible region of the sample (Campbell et al., 2012). However, BIM and the overall sample flexibility may as well affect negatively the quality, the resolution, and the biological interpretation of the three-dimensional cryo-EM reconstructions (Rawson et al., 2016).

A major contribution to the cryo-EM revolution was provided by DEDs, making a huge leap in quality compared to previously available technologies (Grigorieff, 2013). These new detectors can capture electrons directly, without any intermediate conversion steps (Faruqi and Henderson, 2007), resulting in outstanding imaging quality at high resolution, superseding CCD, and even photographic film (Fromm et al., 2015). The dramatic improvement in readouts enabled recording of cryo-EM images in “*movie mode*,” with many frames constituting the final micrograph recorded from a single exposure (Campbell et al., 2012; Li et al., 2013). This allowed tracking of single particles inside each electron micrograph, with better evaluation and compensation of molecular motions due to BIM (Brilot et al., 2012), more careful evaluation of radiation damage (Baker and Rubinstein, 2010; Fromm et al., 2015), and selective classification of multiple conformations within the imaged particles (Bai et al., 2013, 2015a,b; Schwander et al., 2014; Frank and Ourmazd, 2016). This last feature allows multiple reconstructions from a one single cryo-EM dataset, from which different conformers of the same molecule can be obtained (Rawson et al., 2016). These features are critical for reaching the final sub-nanometer high resolutions in recent 3D reconstructions and inspect molecular motions (Campbell et al., 2012; Bai et al., 2013; Li et al., 2013).

Next to hardware improvements, the continuous evolution of cryo-EM imaging softwares, with highly efficient semi- or fully-automated tools for particle picking (Tang et al., 2007; Langlois et al., 2014; Scheres, 2015), motion correction (Li et al., 2013; Rawson et al., 2016), 3D reconstruction (Elmlund et al., 2008; Singer and Shkolnisky, 2011; Scheres, 2012; Brown et al., 2015), 3D structure fitting (Topf et al., 2008; Wriggers et al., 2010; Barad et al., 2015; Brown et al., 2015), and validation using objective criteria (Zhang et al., 2008; Murray et al., 2013) further accelerated the march of cryo-EM in structural biology. Outstanding achievements of cryo-EM showed that this investigation approach is also highly suitable for membrane proteins (Allegretti et al., 2015; Cleverley et al., 2015; Gao et al., 2016), a notoriously challenging field in structural biology. In this respect, cryo-EM enabled studies of integral and membrane-anchored macromolecular systems in more physiological environments than detergent micelles or lipidic-cubic phases typically used in X-ray diffraction experiments. Recent cryo-EM reconstructions of transmembrane proteins reconstituted in nanodiscs (soluble nano-scale phospholipid bilayers constrained by lipoprotein boundaries) showed that the strong electron density for the phospholipid head groups can be efficiently distinguished from the weak density of the region occupied by the acyl chains of the fatty acids, facilitating particle picking, reconstruction and subsequent structural analysis (Frauenfeld et al., 2011; Gao et al., 2016).

### Studying flexible systems using modern cryo-EM

Comparative analyses of crystal and cryo-EM structures of the same macromolecular system are starting to provide clear insights into functionally-relevant features and unprecedented molecular motions thus far concealed by the conformational sampling forced by packing inside crystal structures. Relevant examples are the novel co-receptor site identified in the cryo-EM reconstruction of adeno-associated virus-2 in complex with its receptor and heparin (O'Donnell et al., 2009), or the horizontal transmembrane alpha helices assisting dimerization in the F-type ATP synthase (Allegretti et al., 2015). In both cases, these regions are critical for the biological functions of these molecules, and were never observed in previously determined crystal structures. Likewise, the recent cryo-EM structure of the *E. coli* 70S ribosome in complex with EF-Tu and tRNA enabled identification of new rRNA modifications, not observed in any of the higher resolution ribosome X-ray structures available, because of their flexibility (Fischer et al., 2015).

**Figure 2:**
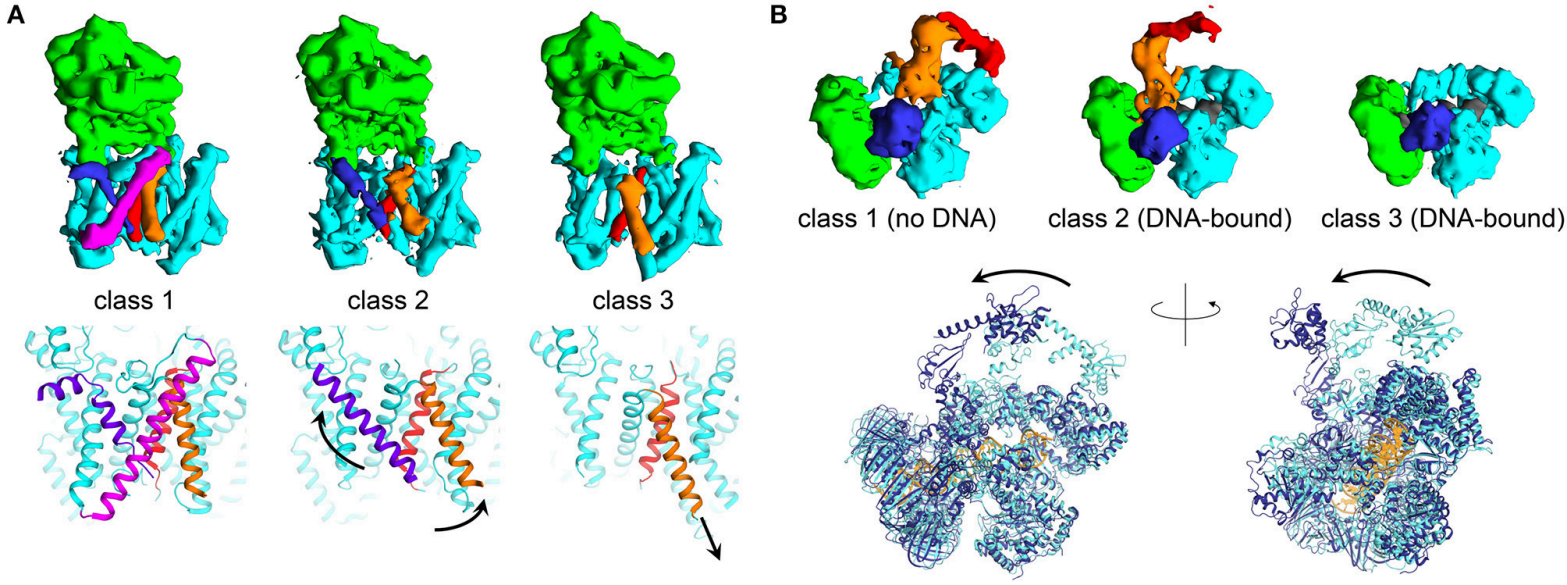
**Trapping multiple conformations using modern cryo-EM. (A)** Three different EM maps obtained from selective classification of the apo gamma secretase cryo micrographs show conformational changes in the transmembrane region of the enzyme complex. Shown are the experimental maps and the three-dimensional structures (obtained from EMDB maps 3238, 3239, 3240, and PDB IDs 5FN3, 5FN4, 5FN5, respectively, Bai et al., 2015b) with soluble nicastrin depicted in green, and the transmembrane region composed of Aph-1, PS1, and Pen-2 components in cyan. Transmembrane helices found in different conformations in the three different classes are shown in blue, red and orange. Arrows indicate the putative movements associated to the rearrangements of the transmembrane helices. **(B)** Three EM reconstructions relative to identification of multiple conformations in DNA-free and DNA-bound *E. coli* PolIIIα-clamp-exonuclease-τ_c_ micrographs (Fernandez-Leiro et al., 2015). PolIIIα is depicted in cyan, the clamp is shown in green, the exonuclease domain is in blue. DNA is colored in dark gray and is present only in classes 2 and 3. The moving regions, composed of the PolIIIα-tail and τ_c_, are shown in orange and red, respectively (data from EMDB maps 3201, 3198, and 3202). The superposition shows the comparison between the structural models obtained from the DNA-free (class 1) and DNA-bound (class 2) states, shown as cartoon and colored in light and dark blue, respectively (PDB IDs 5FKU and 5FKV). DNA for the bound state is shown in gold. Figure prepared using *Chimera* (Pettersen et al., 2004).

There are numerous examples elucidating the ability of cryo-EM to enable direct analysis of conformational changes in large macromolecular complexes. The structure of the complex of human gamma secretase was determined by implementing new structural refinement methodologies, allowing to “focus” the refinement on a defined region of the protein complex of interest. Such strategy allowed overcoming the issue of structural heterogeneity within the cryo-EM dataset, and allowing characterization of atomic features and side-chain allosteric rearrangements in the active site. The same structural refinement methods enabled understanding how inhibitors of the enzyme complex induce conformational rigidification (Bai et al., 2015b; Figure 2A). In a recent study focusing on processivity in cytoplasmic dynein, cryo-EM showed a wide range of conformations, providing for the first time evidence for extensive flexibility to be essential to the function of this molecular motor (Imai et al., 2015). Recently, five ribosome structures in complex with the viral internal entry sites (IRES) and translocase eEF2 were obtained by accurate classification and particle analysis from a single cryo-EM dataset. These structures, refined to maximum resolutions ranging from 3.5 to 4.2 Å, revealed how the viral molecule progressively translocates in a cap-independent manner from the A to the P sites of the ribosome, and provided an unprecedented view of EF2 dynamics (Abeyrathne et al., 2016). Other fascinating examples of the possibilities of cryo-EM in investigating molecular flexibility are provided by the *E. Coli* PolIIIα-clamp-exonuclease-τ_c_ complex and the hexameric AAA ATPase p97. In the 8 Å resolution structures of DNA-bound and DNA-free states of the PolIII-replisome complex, even if nearly all the proteins composing the complex are flexible enough to hinder crystallography, the cryo-EM structures clearly revealed conformational changes critical for interaction of the replisome with DNA (Fernandez-Leiro et al., 2015; Figure 2B). The cryo-EM micrographs of the hexameric AAA ATPase p97 showed three distinct, co-existing functional states of p97 with differential ATP occupancy per protomer, accompanied by large rearrangements of structural elements in the ATPase fold. Interestingly, the conformations obtained in the cryo-EM reconstructions were never observed in the crystal structures of p97. This example illustrates how multiple 3D reconstructions of distinct conformations of a dynamic macromolecule can be obtained from a single cryo-EM dataset by accurate particle selection and classification after particle picking (Banerjee et al., 2016).

Still, the most remarkable example of how cryo-EM is dramatically changing all structural biology paradigms, is perhaps the very recent structural characterization of small (<100 kDa) enzymes in complex with small-molecule inhibitors (Merk et al., 2015). Remarkably, a single paper experimentally summarizes the outstanding potential of cryo-EM in investigating molecular flexibility. By breaking the 2.0 Å resolution limit and challenging macromolecule sizes below 100 kDa (also thanks to application of the latest phase plate technologies), the authors did not simply demonstrate that cryo-EM is suitable for drug discovery and structural enzymology, but also provided for the first time clear details about molecular allostery mediated by binding of inhibitors (Merk et al., 2015). Such a remarkable result possibly sets the starting point for a new era of structural analysis using cryo-EM, with biological outcomes that even at present are not completely imaginable.

## Conclusions

**Figure 3:**
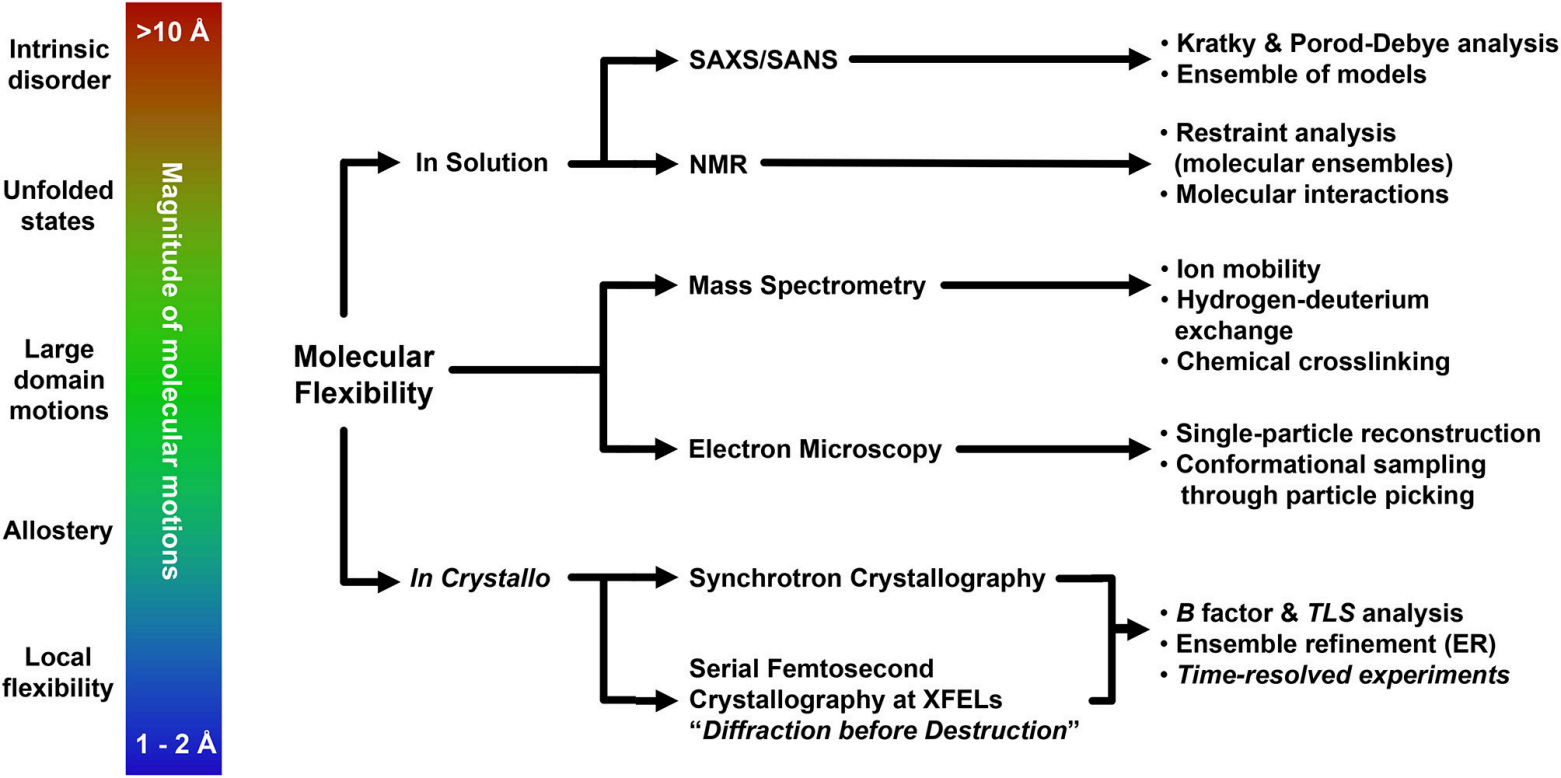
**Representative flowchart addressing modern experimental structural biology approaches for the understanding of molecular flexibility**.

Conformational flexibility is the driving force of a plethora of biological events, and understanding the contributions of dynamics to macromolecule function is a fundamental aspect of basic and applied biological research. Over the course of this review we have described how several cutting-edge structural biology techniques may provide a broad toolbox to explore molecular flexibility, with emphasis on the possible outcome of the investigation and on the methodological approaches to employ. The choice of the most appropriate experimental strategy to carry out the investigation must take into account the overall extent of conformational changes, and will likely involve the usage of multiple structural biology methods (Figure 3). Given the complexity of these studies, it is natural that additional experimental validation using biophysics or other specific methods is of paramount importance. This holds especially true for low resolution methods, which nonetheless can be fundamental for preliminary investigation as well as solid sources of corroborating data, as shown by the usage of SAXS (Hura et al., 2009; Pelikan et al., 2009; Rambo and Tainer, 2011; Bernadó and Svergun, 2012; Hammel, 2012; Petoukhov and Svergun, 2013; Dyer et al., 2014; Kachala et al., 2015) but possibly also by novel, unorthodox methodologies that may provide unexpected, remarkable results (Longchamp et al., 2012, 2016). Although the promise of single-molecule structural biology remains far from possible at the moment (Henderson, 2002; Fratalocchi and Ruocco, 2011), serial femtosecond crystallography at XFELs (Martin-Garcia et al., 2016), as well as atomic resolution single-particle cryo-EM (Bai et al., 2015a; Merk et al., 2015; Subramaniam et al., 2016) are now reality. Combined with more “conventional” structural approaches, these techniques nowadays enable extrapolation of relevant structural information also from datasets so far considered untreatable (Hollenstein et al., 2014; Murray et al., 2016), pushing the resolution limits (Karplus and Diederichs, 2012; Lang et al., 2014; Merk et al., 2015) and further bridging the gap between molecular and cellular approaches of biological investigation (Schröder, 2015; van den Bedem and Fraser, 2015).

A converging aspect of the various approaches discussed in this review concerns the final readout generated by the investigation. Most methods generate structural ensembles (Levin et al., 2007; Rambo and Tainer, 2010; Burnley et al., 2012; Schwander et al., 2014; Clark et al., 2015; Keedy et al., 2015; Urzhumtsev et al., 2015; Van Benschoten et al., 2015; Abeyrathne et al., 2016), explicating the information about molecular flexibility through uncorrelated, superimposed conformations that should be analyzed as a whole. There is a need for reliable tools to efficiently compare and visualize complex ensemble data with the same efficiency and user-friendliness of traditional softwares for superpositions and structural comparisons. Computational methods to perform such analyses on large ensembles are still quite limited, and the first truly useful tools are just becoming available (Burnley and Gros, 2013; Clark et al., 2015; Varadi and Tompa, 2015). It is expectable that development of efficient methods of analysis applied to structural ensembles will proceed with the same pace of the methods that experimentally generate those ensembles from structural data. This will be essential to facilitate usage and dissemination of the insights gained from structural analysis of flexible systems. Similarly, advanced computational tools for structural bioinformatics such as structure prediction, molecular docking and *in silico* directed evolution should facilitate the integration of large ensemble data inside their routines, to further expand the capabilities of integrative experimental and computational approaches.

We are confident that the novel pioneering achievements reached by the structural biology community over the last years will pave the way to a future where accurate description of molecular motions will be more and more an integral part of every molecular model. These developments will facilitate the understanding of fundamental biological mechanisms and will speed up also other computational and biophysical methods (such as for example *in silico* drug discovery and protein engineering) that rely on accurate experimental data on macromolecular recognition mechanisms, allostery and conformational variability.

## Author contributions

All authors listed, have made substantial, direct and intellectual contribution to the work, and approved it for publication.

### Conflict of interest statement

The authors declare that the research was conducted in the absence of any commercial or financial relationships that could be construed as a potential conflict of interest.
